# The synergistic effect of artificial intelligence technology in the evolution of visual communication of new media art

**DOI:** 10.1016/j.heliyon.2024.e38008

**Published:** 2024-09-17

**Authors:** Yan Zhao

**Affiliations:** a Faculty of Innovation and Design, City University of Macau, Macau, 999078, China; b School of Art and Design, Guangzhou Vocational College of Technology & Business, Guangzhou, 511630, China

**Keywords:** Artificial intelligence, New media, Convolutional neural network, Visual communication, Design logic

## Abstract

This study aims to clarify the synergistic effect of artificial intelligence (AI) technology in the evolution of visual communication of new media art, thereby exploring an AI layout design method based on Convolutional Neural Network (CNN) in the practice of visual communication design. Firstly, this study designs an AI layout design model based on CNN, and trains and optimizes it with training data. Secondly, the automatic generation of layout design is realized by constantly adjusting the model parameters and network structure. Finally, various AI layout design algorithms are compared, and their effects and performances in layout design generation are analyzed. To verify the layout and composition matching model's performance, traditional layout design methods are selected for comparison (layout, comparison, harmonic composition, etc.). This study involved 20 design students as participants, evaluating them across three dimensions: overall comprehensive assessment, readability of text information, and rationality of visual path using a Likert 7-point scale. The results reveal that the proposed method's evaluation outcomes in these three aspects are 5.95, 5.68, and 5.74, respectively, higher than the traditional layout design methods. To sum up, the generative AI discussed here can automatically generate design elements and schemes through deep learning and big data analysis, thus providing a reference for the innovation of visual communication design.

## 1 Introduction

### 1.1 Research background and motivations

With the rapid advancement of science and technology, artificial intelligence (AI) is increasingly permeating every facet of society, especially in the realm of visual communication within new media art. Its influence and potential applications are growing significantly [[1], [2], [3]]. New media art, an innovative fusion of digital technology and artistic creation, is reshaping aesthetic perceptions and cultural experiences in unprecedented and innovative ways. Exploring the synergistic impact of AI technology on the evolution of visual communication in new media art holds immense theoretical value and offers vast opportunities for practical application [4]. At present, there are more and more studies on the combination of AI and art design, which reveal the potential of AI technology in art creation and design from different angles. Choi et al. (2021) [5] analyzed the application of deep learning (DL) algorithms in image generation to demonstrate how AI could simulate and surpass certain aspects of traditional art creation; Zhao et al. (2024) [6] discussed the role of AI-aided design system in improving design efficiency and creativity, emphasizing the importance of human-machine collaboration.

Visual communication design in new media art is a multifaceted field that encompasses graphic design, color theory, typographic principles, and other disciplines [7,8]. Graphic design ensures effective information transmission and precise emotional expression through composition principles and the symbolism of graphic elements. Color theory underscores the pivotal role of color in visual communication, with different colors evoking distinct emotional associations and experiences. Typographic principles involve organizing text, graphics, and other elements to create clear and organized visual presentations. These elements collectively form the foundational and theoretical system of visual communication design in new media art [[9], [10], [11]].

The development of AI technology has revolutionized visual communication design in new media art. As a critical branch of intelligence science and technology, AI, with its robust capabilities to simulate, extend, and enhance human intelligence, offers limitless possibilities for visual communication in new media art [[12], [13], [14]]. AI enables real-time interaction between the audience and artworks. Through speech recognition and synthesis technology, AI can make the audience have a dialogue with the works of art. Additionally, it can analyze the audience's behavior and preferences through machine learning (ML) algorithms to provide personalized interactive experiences. The application of ML, DL, natural language processing (NLP), computer vision, and other technologies in new media art makes visual communication design more intelligent, personalized, and efficient. AI can accurately predict and recommend user preferences by leveraging ML algorithms to analyze vast amounts of data [15]. DL technology plays a pivotal role in image recognition, speech recognition, and related fields, enhancing the accuracy and efficiency of visual communication. NLP enhances human-computer interaction (HCI), making it more natural and seamless, thereby bringing new creative inspirations and expressive forms to new media art. However, as AI technology progresses, concerns arise about the potential displacement of human artists. This apprehension raises ethical questions about employment, social status, and identity. It is crucial to explore how to ensure the livelihood and development opportunities for human artists while advancing technological progress.

Algorithms play a crucial role in new media art, offering creative inspiration and aiding decision-making for artists through the processing and analysis of extensive data. ML-based algorithms can extract specific patterns from vast image datasets, audio, and video, empowering artists to generate new artworks. Moreover, algorithms can dynamically adjust the expression of artistic works based on audience feedback and behavioral data to deliver personalized experiences. System design is equally integral to new media art, with outstanding systems needing high customizability and scalability to meet diverse artist and audience needs. During system design, considerations such as user experience, interaction modes, data processing capabilities, and more must be thoroughly addressed.

Although existing studies have revealed many possibilities of AI in the art design field, there are still deficiencies in the discussion of the specific synergistic effects and realization mechanisms of AI in the visual communication design of new media art, especially layout design. Especially CNN, as one of the representative technologies of DL, its excellent performance in image recognition and processing provides the possibility for the automation and intelligence of layout design, but the related practice and research still need to be in-depth. The main objective of this study is to offer new ideas and directions for research and practice in related fields by deeply exploring the synergistic effect of AI technology in the evolution of visual communication of new media art. Meanwhile, this study aims to promote the further integration and development of new media art visual communication and AI technology, contributing to cultural innovation and industrial upgrading.

### 1.2 Research objectives

The research objective of this study is to fully reveal the synergistic effect of AI technology in the evolution of visual communication of new media art. It aims to delve deeply into the specific application of this technology in visual communication design practice and the AI layout design method based on Convolutional Neural Network (CNN). This study aims to provide new ideas and directions for the innovation and development of visual communication in new media art.

Section I describes the research background, objectives, and research necessity. Section II summarizes the related research status of new media art and visual communication design supported by AI. Section III mainly analyzes the application of generative AI in visual communication, and a CNN-based intelligent layout design method is proposed for the new media art design carrier. Section IV presents the experimental design and analysis, completing the evaluation of the intelligent layout design method for visual communication. Section V summarizes the research contribution, limitations, and future research direction.

## 2 Literature review

### 2.1 Research status

New media art is a form of artistic creation and display characterized by digital technology, interactivity, and real-time features. Compared with traditional art, it pays more attention to the audience's participation, interaction, and feedback, breaking the boundaries of time and space. Hou et al. (2022) [16] elaborated on the application of virtual reality (VR), augmented reality (AR), and interactive three-dimensional (3D) technology in art exhibitions and cultural heritage protection. Their study deeply analyzed how these technologies enhance audience participation and experience, and discussed their potential in cultural inheritance and education. Sun et al. (2022) [17] examined the profound influence of AI technology on the artistic creation process and the presentation form of works focusing on the relationship among artists, audiences, and markets. They highlighted the role of ML and Generative Adversarial Net (GAN) in promoting artistic innovation. Gong et al. (2022) [18] reviewed the development trend of AI in the art and culture fields, analyzing the far-reaching impact of AI technology on artistic creation and display. They explored dimensions such as computational creativity, automatic creation, artistic creation, and HCI. Shi et al. (2023) [19] emphasized the complementary advantages of collaboration between designers and AI in the design process, guiding HCI research and practice to focus more on collaborative creativity. Song et al. (2024) [20] explored AI-based intelligent application design and adaptive interface design, with practical applications in intelligent recommendation systems, voice assistants, and search engines. Wang (2024) [21] proposed a layered model that decomposed images into pixel, element, relationship, plane, and application layers, defining feature representations for each category. The model used clustering algorithms to extract element colors, calculate layout and color perception features, and implement a predictive model for image geometric features. The model was used to predict the probabilistic distribution of position and color elements under uncertain conditions to facilitate the creation of graphic design works by fitting the geometric feature distribution found in the data. Finally, the study reconstructed graphic designs based on interactive characteristics, generating diverse graphic design images. Zhan et al. (2024) [22] constructed an integrated intelligent optimization of a Computer-Aided Design (CAD) system by adopting a CAD requirement analysis method based on media big data and an integrated strategy of intelligent optimization algorithm. The system was capable of automatically acquiring and processing media big data and automatically optimizing and iterating CAD schemes using intelligent optimization algorithms. The experimental results fully prove that the design using this method significantly improved in terms of innovation, practicality, and aesthetics. The system could effectively use media big data to guide design decisions, improve quality, and reduce manual intervention and design iteration time. Gu et al. (2023) [23] proposed to build an AI-based visual communication system. In this way, the image could be made clearer, with a larger field of view and magnification. At the same time, the system was applied to modern art design, which was an innovation, and the system had great advantages in graphic conversion. In addition, the color difference of the optical system could be corrected to improve the imaging effect.

Interactive experience technology supported by AI mainly relies on advanced technologies such as AI algorithms and ML to realize real-time and intelligent interaction between people and new media artworks. Zhao et al. (2024) [24] pointed out that AI technology could intelligently extract and combine design elements through ML and big data analysis, enabling the automatic generation of design works. Meanwhile, AI can also make personalized recommendations and optimize the design according to users' preferences and needs, enhancing the pertinence and effectiveness of the design. Ai (2019) [25] explored the innovative practice of visual communication design based on AI. By introducing technologies such as the DL algorithm and CNN, intelligent analysis and optimization of design works were realized. Karlsson Häikiö (2022) [26] believed that the introduction of AI could greatly simplify the design process and improve design efficiency. AI can also provide designers with more creative inspiration and ideas, fostering the innovative development of visual communication design. Chiu et al. (2024) [27] examined the application of AI technology in art education, particularly through AI-assisted art learning systems using DL technology. This system utilized a fine-tuned ResNet50 model to help students recognize and classify artworks, aiming to develop their accurate appreciation and creative abilities while providing instant feedback and personalized guidance through AI technology. Taye (2023) [28] investigated the application of CNN-based image recognition technology in visual art creation, finding significant advantages in improving creative efficiency and design precision. Additionally, Wingström et al. (2024) [29] researched the innovative applications of AI in new media art creation, particularly how big data analysis and DL algorithms could predict and generate the visual effects of artworks. The study highlighted that AI technology not only enhanced creative efficiency but also expanded the boundaries of visual design through data-driven approaches. Chung et al. (2023) [30] proposed a GAN-based design model for generating dynamic visual effects in new media art, demonstrating its contribution to the diversity and innovation of visual design during the creative process.

### 2.2 Research review

In summary, existing research has extensively explored the applications of AI technology in new media art, particularly its potential in visual communication design. Overall, the literature primarily focuses on AI's role in assisting the artistic creation process, improving design efficiency, and enhancing audience interaction experiences. AI technologies, such as CNNs and GANs, have been employed in tasks like image recognition, layout optimization, and automatic generation of design elements, markedly improving creative efficiency and design precision. Moreover, intelligent recommendation systems, as well as VR and AR technologies, have been applied in art exhibitions and cultural heritage preservation, enhancing audience engagement and experience. However, these studies often emphasize the technical application aspects, lacking a systematic exploration of the deep integration of AI and visual communication design. Additionally, when assessing the actual effectiveness of AI technology, current research tends to focus on a single dimension, neglecting multi-dimensional comprehensive evaluations and comparative analyses. In contrast, this study presents a CNN-based AI layout design model, aimed at achieving deep application and optimization of AI technology in visual communication design. Through continuous adjustment of model parameters and network structures, this study achieves automated layout generation and conducts comparative analyses of various AI layout design algorithms, thereby validating their effectiveness and superiority in actual design tasks. The innovation of this study lies in adopting a multi-dimensional comprehensive evaluation approach, particularly by systematically verifying the AI layout design model's advantages in design effectiveness, text readability, and visual path rationality, compared to traditional design methods. This provides strong support and reference for the innovation of visual communication design and the deep integration of AI technology.

## 3 Research methodology

### 3.1 Application of generative AI in visual communication

**Fig. 1:**
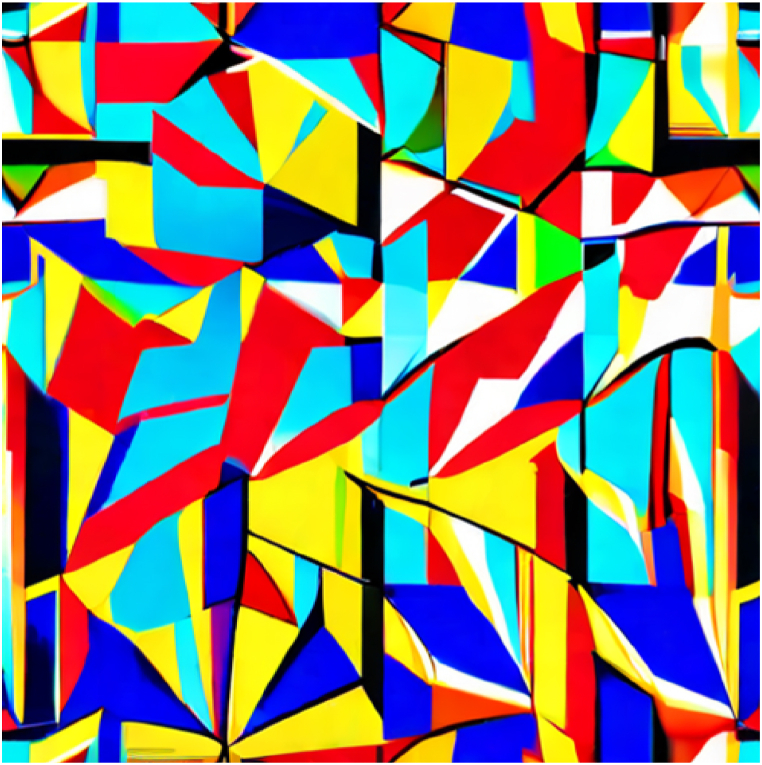
Artistic works generated by generative AI.

From a design perspective, generative AI can automatically generate design elements and schemes through DL and big data analysis. It can learn and analyze vast amounts of images, characters, sounds, and other data based on preset algorithms and models, extracting key information and generating creative designs [[31], [32], [33]]. In contrast, traditional art design focuses more on the creativity and manual skills of designers, who need to accumulate extensive design experience and aesthetic quality through long-term practice and study to create works with unique style and connotation. Fig. 1 displays the works of art generated by generative AI.

While AI itself is not capable of subjective emotion and creativity, it can simulate the human creative process by analyzing a large number of artworks, design principles, and aesthetic rules. AI systems are trained to recognize and understand artistic elements of different styles, themes, and emotional expressions, thereby generating a “database” of ideas within themselves. AI is also good at style transfer, where the style of one work of art is applied to the content of another. By analyzing the stylistic characteristics of different artworks, AI can generate artworks with mixed styles or entirely new styles, creating unprecedented visual experiences.

Generative AI offers significant advantages in terms of design efficiency. It can rapidly produce multiple design concepts and adjust them in real-time based on user feedback and requirements. This streamlined design process significantly shortens design cycles and enhances overall efficiency. Traditional art design often requires more time and effort to conceive and produce works, particularly for complex design tasks where efficiency may be more constrained.

The core of generative AI lies in its capability to learn and generate novel content from extensive datasets [34]. In the field of visual communication, this ability is manifested in the intelligent processing of design elements such as graphics, colors, and layout [[35], [36], [37]]. Table 1 exhibits the application of generative AI in visual communication.

**Table 1:** Application of generative AI in visual communication.

Application area	Application content	Technical introduction	Advantages
Image production	Automatic image design	GAN, CNN, and other DL algorithms	Efficient, innovative, and customizable
Color matching	Intelligent recommendation color scheme	Based on color theory and user data	Improve design consistency and meet user needs
Graphic design	Generate innovative graphic elements	Learn based on graphic library and algorithm	Innovation and diversity
Format design	Automatic typesetting and layout	ML algorithm and rule engine	Improve efficiency and consistency
Video production	Automatically generate video clips and special effects	Learn based on video library and algorithm	Efficient and innovative
Virtual reality	Automated scene generation and rendering	3D modeling and rendering algorithm	Quickly build a virtual environment
Advertising design	Automated advertising creativity and typesetting	Based on advertising data and user feedback	Improve the advertising effect and conversion rate

Taking CNN as an example, it can extract the key features from massive image data and generate brand-new and unique design elements based on it. Specifically, CNN operates by progressively extracting features from input images through multiple convolutional layers. Each convolutional layer employs numerous kernels that traverse the image, capturing and extracting diverse visual features. With the deepening of network layers, the extracted features become increasingly abstract and sophisticated. This feature extraction process from concrete to abstract enables CNN to accurately capture the key information in the image and generate new design elements based on this extracted knowledge.

In visual communication design, generative AI's ability can be applied to many aspects. For example, in color matching, generative AI can intelligently recommend and generate color-matching schemes that align with specific styles and emotional colors according to user preferences and design requirements. In graphic design, AI can create innovative and artistic new graphics by analyzing and learning existing graphics [[38], [39], [40]]. Furthermore, generative AI extends its utility to fields such as layout design and font design, offering designers a broader spectrum of creative options.

Beyond its role in processing design elements, generative AI excels in real-time optimization through continuous user feedback and data analysis. By gathering and interpreting user data during interactions, AI can discern preferences and behaviors, thereby refining design outputs to better align with users' expectations and requirements.

### 3.2 Conversion between design logic and calculation logic in visual communication

Design logic primarily concentrates on the conception, planning, organization, and optimization processes of products or systems. It emphasizes human creative thinking, needs analysis, functional layout, aesthetic considerations, and overall enhancement of user experience. In design logic, designers use methods such as hand-drawn sketches, model-making, and prototype testing to transform abstract concepts into tangible, perceptible forms. Design logic highlights a human-centered design philosophy, aiming for convenience, comfort, and satisfaction during the use of products or systems. In contrast, computational logic relies more on computer technology and algorithmic support. It focuses on translating the concepts and plans from design logic into executable sequences of computer instructions. In computational logic, programmers or algorithm designers use programming languages, data structures, and algorithm design tools to translate functional requirements, performance metrics, and constraints from design logic into computational tasks that computers can understand and execute. Computational logic emphasizes precision, efficiency, and scalability, striving for accuracy, speed, and flexibility in data processing by computer programs.

Based on requirements analysis, mathematical models or logical models are constructed for products or systems. These models describe the internal structure and behavioral characteristics of products or systems, guiding the implementation of computational logic. According to the results of model construction, corresponding algorithms are designed to realize the functions of products or systems. Algorithm design considers factors such as correctness, efficiency, and stability to ensure that computational logic accurately implements the requirements of design logic.

Images and graphics are among the most basic elements of visual communication. They intuitively display features such as the objects' shape, color, and texture, conveying specific information and concepts. Colors play a crucial role in visual communication. Different colors can evoke various emotional responses and associations, influencing people's emotions and attitudes. The choice of layout and fonts significantly impacts the effectiveness of visual communication. Proper layout can make information clearer and easier to read, while suitable fonts can convey specific styles and atmospheres. In the context of AI, the design logic in visual communication of new media art needs to be transformed into computational logic, enabling machines to understand and perform design tasks [41]. The transformation involves moving from abstract, intuitive concepts to concrete, rational processes.

Firstly, it is necessary to analyze and refine the designer's design ideas and goals and turn them into languages and rules that computers can comprehend. This usually involves defining design elements, quantifying design principles, and standardizing design processes. Secondly, AI algorithms and models are employed to convert these design logics into computational logics. This includes using an ML algorithm to learn and simulate the designer's creative process, optimization algorithms to find the optimal solution that meets the design objectives and constraints, and generative models to produce design schemes that satisfy design requirements.

**Fig. 2:**
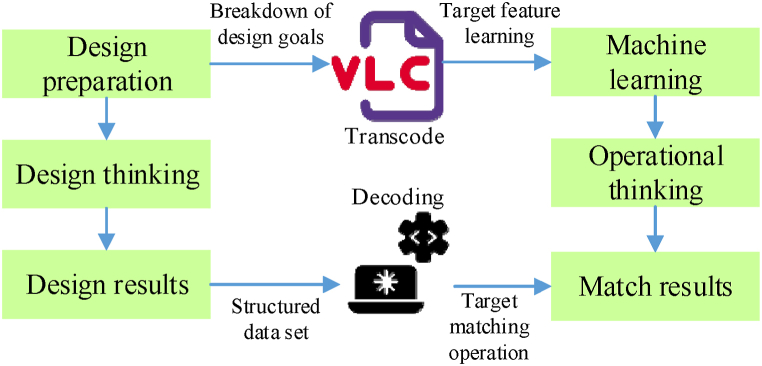
Transformation from design logic to computational logic.

In this process, data plays a vital role. By collecting and analyzing extensive design data, people can better understand the nature and laws of design logic, and then optimize the implementation of computational logic [[42], [43], [44]]. Furthermore, data feedback continuously enhances and refines both design and computation logic, thereby enhancing design efficiency and quality. Once the transformation from design logic to computational logic is complete (Fig. 2), AI can autonomously generate design schemes that adhere to specified requirements and constraints.

Layout composition is an important part of design, which involves many aspects such as element layout, space allocation, and color matching [[45], [46], [47]]. Different forms of layout composition can convey diverse visual effects and emotional atmospheres. Therefore, in-depth study and understanding of layout composition is essential to achieve accurate matching.

**Fig. 3:**
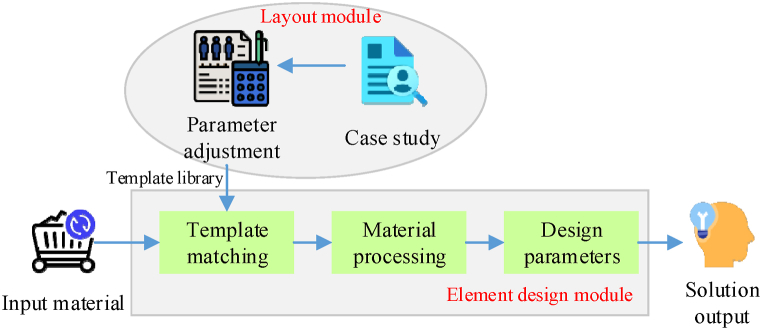
Matching model architecture based on layout composition.

The matching model based on layout composition first analyzes the design works to extract the layout composition elements, such as words, graphics, and colors. Then, these elements are transformed into quantifiable feature vectors or feature matrices for mathematical calculation and comparison. In the matching process, the model compares the similarity of feature vectors or feature matrices of layout composition among different design works using algorithms like cosine similarity and Euclidean distance [48,49]. According to these algorithms, the model can accurately judge the similarities and differences of various design works in layout and composition. The matching model architecture based on layout composition is presented in Fig. 3.

### 3.3 Layout design of AI based ON CNN

Through the convolutional layer, pooling layer, fully connected (FC) layer, and other structures, CNN abstracts and extracts the features of the input image layer by layer, and finally outputs the classification or recognition results of the image [[50], [51], [52]]. In the convolutional layer, multiple convolution kernels are employed to convolve the input data, extracting feature maps at different levels. These feature maps contain important visual elements and structural information crucial to layout design. The pooling layer is responsible for pooling operations (such as max pooling, average pooling, etc.) on the feature maps output from the convolutional layer, reducing their dimensions and computational load while preserving vital feature information. Through the alternating stack of multiple convolutional and pooling layers, the feature extraction layer gradually extracts deep features specific to layout design. In the design element matching layer, features extracted by CNNs are used to identify and match design elements (such as text, images, colors, etc.) within the layout. By constructing matching algorithms, optimal combinations and layout optimizations of design elements are achieved [53]. Key parameters in layout design (such as element positions, sizes, spacings, etc.) are parameterized. These parameters serve as optimization variables in the differential model, adjusting them to optimize the layout. During optimization, various constraints of layout design, such as readability, aesthetics, functionality, etc., need to be considered. These constraints can be transformed into constraint functions within the differential model, combined with the loss function using methods like Lagrange multipliers, forming a complete optimization problem [54]. In layout design, design elements such as words, graphics, and colors can be regarded as pixels of an image, and CNN can be used for feature extraction and learning. Table 2 outlines the layout design process of AI based on CNN.

**Table 2:** AI layout design process based on CNN.

Step	Process content	Description and operation
1	Data collection and pretreatment	A large number of layout design samples are collected, encompassing various design styles, element combinations, and layout methods. The collected data undergoes preprocessing, such as resizing images, converting color spaces, normalization, etc.
2	Construction of the CNN model	The CNN model structure is designed, determining parameters of the model, convolution kernel sizes, strides, etc. Appropriate activation functions, loss functions, and optimization algorithms are selected.
3	Model training and optimization	The preprocessed training set is used to train the model, updating model parameters using the backpropagation algorithm. The model's performance during training is monitored, adjusting model parameters based on performance metrics.
4	Model evaluation and optimization	The test set is utilized to evaluate the trained model, calculating performance metrics. Based on evaluation results, further tuning of the model is conducted.
5	AI layout design generation	New design elements are input into the trained CNN model. The model extracts features from the input elements and analyzes their layout, generating layout design results based on learned design patterns.
6	Results display and adjustment	The generated layout design results are presented to the user. Input elements or parameters are adjusted based on user feedback, iteratively generating new design proposals.

The convolutional layer is the core part of CNN, which is used to extract the features of input images. In the panel design model, multiple convolutional layers are employed, each containing numerous convolution kernels that slide across the input image to capture local features. As the network deepens, these convolutional layers can learn more abstract and complex features. Positioned after the convolution layers, the pooling layer downsamples the output from the convolutional layer to reduce data dimensionality and computational load. Max-pooling, a common pooling operation, preserves spatial structure while reducing data dimensions. Following multiple convolutional and pooling layers, the FC layer integrates and classifies the learned features. This layer flattens the feature map output into one-dimensional vectors and maps them to the output space through the weight matrix. The output layer utilizes the Softmax function to classify and generate the probability distribution across each category.

Initially, a large dataset of artistic works and design samples is collected and preprocessed including image scaling, cropping, graying, and other operations. Subsequently, the preprocessed image data is input into a pre-trained CNN for training. Through multi-layer convolution and pooling operations, the network gradually learns the feature information contained within the images. The model is trained using the prepared training data, and its parameters are optimized through the backpropagation algorithm. During training, techniques such as data augmentation and regularization can be employed to prevent overfitting and enhance the model's generalization ability [55]. Post-training, the trained CNN extracts high-level feature representations from the images, which are then utilized to generate a new layout design.

Regarding image cropping, technology based on template constraint adaptation can automatically analyze image materials uploaded by users and intelligently crop them according to predefined size, proportion, and layout requirements. This process includes the picture size's automatic adjustment and the picture content's intelligent identification and cropping.

## 4 Experimental design and performance evaluation

### 4.1 Datasets collection

This study employs a series of sophisticated data analysis techniques and experimental design processes to explore the synergistic effects of AI technology in the evolution of new media art visual communication. First, a meticulously constructed and trained AI model is utilized to validate the effectiveness of the CNN-based AI layout design method. In terms of data collection, 20 students with backgrounds in design or visual communication are invited to participate in the experiment, ensuring gender balance, and a large number of layout design samples in various styles are collected. These data undergo preprocessing steps, including resizing images, converting color spaces, and normalization, ensuring the model can efficiently and accurately learn the features. During model training, batch gradient descent is used to optimize model parameters, with L2 regularization and dropout mechanisms introduced to prevent overfitting, and TensorFlow's GPU acceleration is employed to enhance computational efficiency. Several hyperparameters, such as learning rate, the number of convolutional layers, and kernel size, are set to evaluate the model's performance, with the optimal configuration determined through experimentation. By comparing traditional design methods (such as layout, contrast, and harmonious composition) with AI-generated methods, the results are quantified using a Likert 7-point scale. During testing, participants are divided into two groups, observing posters with a 10-min interval between sessions, with random displays to reduce subjective influence, ensuring fairness and accuracy of the data. In the data analysis phase, multiple mathematical algorithms (such as cosine similarity and Euclidean distance) are applied for an in-depth comparative analysis of different design schemes.

### 4.2 Experimental environment

Processor: Intel Core i9-10900 K, 10 cores and 20 threads, clocked at 3.7 GHz. Memory: 64 GB DDR4 RAM with a frequency of 3200 MHz. Graphics card: NVIDIA GeForce RTX 3080, 10 GB GDDR6X memory.

Operating system: Ubuntu 20.04 LTS, 64-bit. DL framework: TensorFlow 2.1, accelerated by GPU. Data processing library: Pandas 1.3, numpy1.3.

### 4.3 Parameters setting

The number of convolutional layers is 5. The number of convolution kernels in each layer is 32, 64, 128, 256, and 512 in turn. The convolution kernel size is 3 × 3, and the number of neurons in the FC layer is 1024. The activation function is ReLU.

The learning rate is initialized to 0.001, and the exponential decay strategy is adopted. The batch size is 32. The number of iterations is 200 epochs, and the optimizer is Adam.

### 4.4 Performance evaluation

**Fig. 4:**
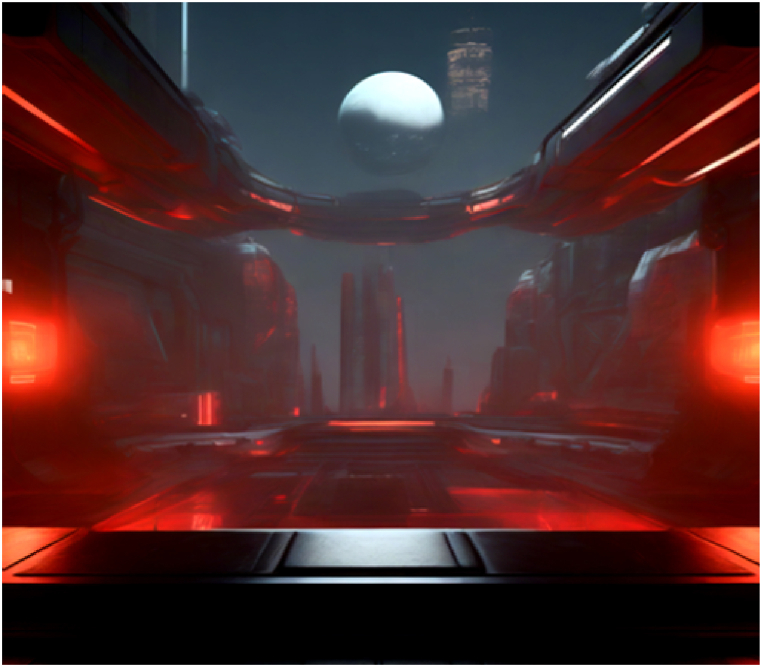
Posters designed based on this research method.

Images in poster design serve not only as visual elements but also convey themes and emotions. In this study, the selection and processing of images in AI-generated posters effectively convey specific emotions and themes, thus enhancing their expressive power. In the designed poster, through the organic combination of images and texts, the information to be expressed by the poster is effectively conveyed, enabling clearer understanding of their content and themes by the audience. The poster designed based on this research method is depicted in Fig. 4.

**Fig. 5:**
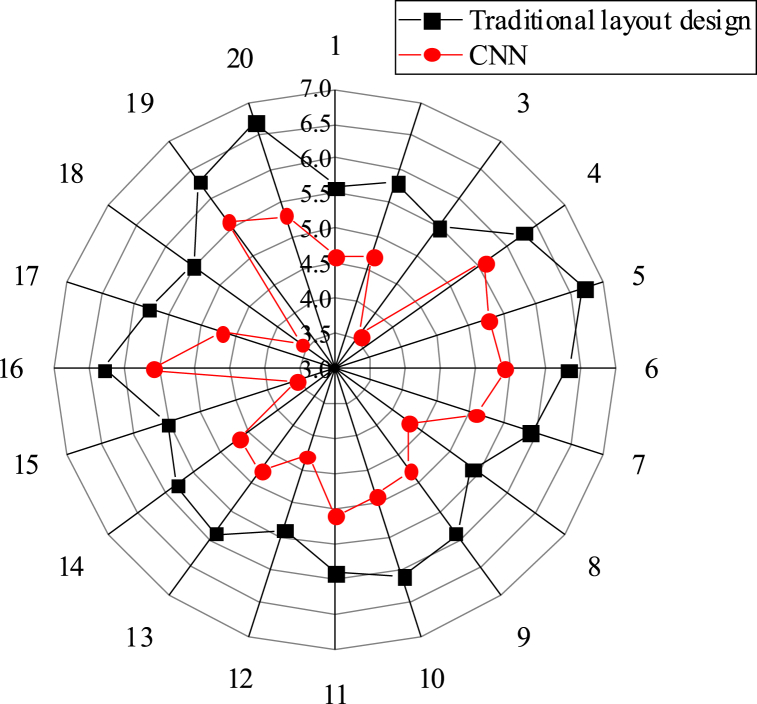
Score statistics of overall comprehensive evaluation.

**Fig. 6:**
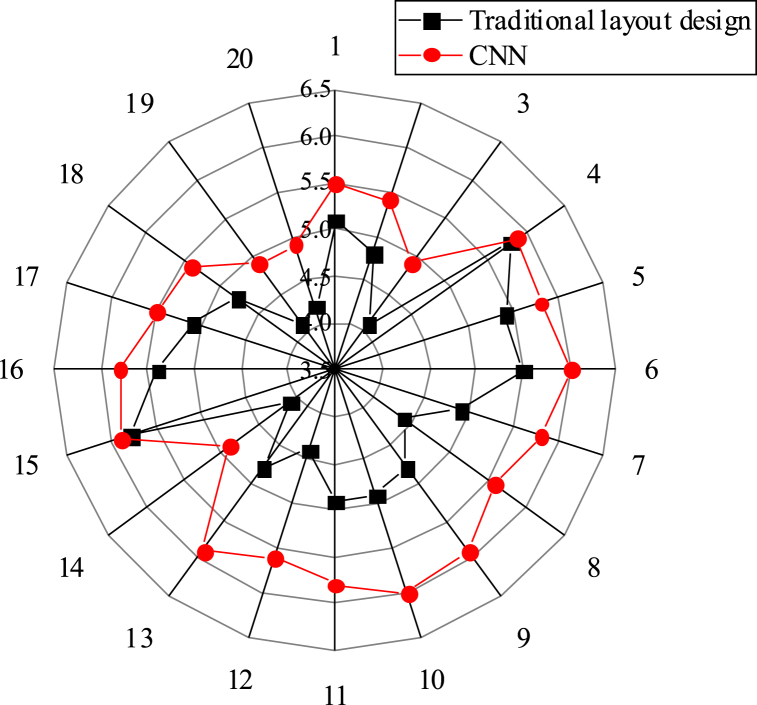
Score statistics of readability evaluation of text information.

**Fig. 7:**
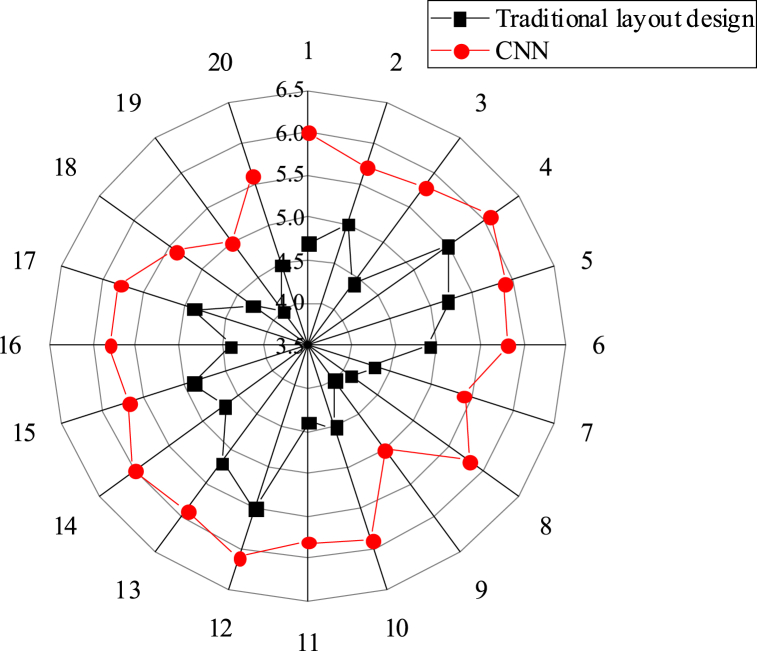
Score statistics of rationality evaluation of visual path.

This study compares the effectiveness of the CNN matching model-based layout design with traditional methods. Fig. 5, Fig. 6, Fig. 7 present the scoring results in three aspects: overall comprehensive evaluation, readability evaluation of text information, and rationality evaluation of visual path. The scores obtained for these aspects are 5.95, 5.68, and 5.74 respectively, all of which surpass those achieved by traditional layout design methods.

To clearly understand the influence of each key component on the proposed method, an ablation experiment is designed in this study. The model achieves an accuracy of 85.6 % on the test set when all components are used. However, upon removing the feature extractor, the accuracy drops notably to 70.2 %. The feature extractor is responsible for extracting features from images and texts. Its absence results in ineffective input features, significantly reducing the model's performance. Several model structures with different complexity are designed and their performances are compared and analyzed. The findings indicate that the model structure with deeper and more intricate connections performs better in the visual communication task of new media art, achieving an accuracy improvement of 11.3 %.

### 4.5 Discussion

New media art includes both traditional art forms such as painting and sculpture, as well as cutting-edge fields such as digital art, interactive art, and VR. Advanced generative AI models can produce realistic images or graphics, which have immense practical value in illustration, icon design, poster creation, and other design aspects. Designers can utilize these technologies to quickly create visual elements, enhancing their design works’ attractiveness and expressive power. Through smart algorithms and ML technologies, generative AI can automate certain design tasks such as layout and color schemes. This enhances design efficiency and reduces the burden of repetitive work, allowing designers to focus more on creative conceptualization and optimizing overall design solutions. Format design plays a pivotal role in the visual communication of new media art, serving not only as a medium for information presentation but also as a crucial avenue for artists and designers to convey their thoughts and emotions. User interaction stands as an integral component of AI-driven design. By offering an intuitive and user-friendly interface coupled with interactive functionalities, users can seamlessly communicate their preferences and requirements to the system. The system, in turn, should autonomously adjust design parameters and outputs based on user feedback, thus catering to individual user needs. This adaptability enhances the practical applicability of AI-driven design methodologies across diverse media and art forms.

This study confirms the synergistic effects of AI technology in the evolution of new media art visual communication, particularly in layout design applications. Based on the evaluation results of the 20 design students, the CNN-based AI layout design model proposed in this study achieves average scores of 5.95, 5.68, and 5.74 in the dimensions of comprehensive evaluation, text readability, and visual path rationality, respectively, all remarkably higher than traditional layout design methods. This indicates that the AI-driven layout design model outperforms traditional methods across multiple dimensions. These findings contrast with existing research. For instance, Wang et al. (2023) [56] derived an average score of around 5.2 from traditional manual design methods based on long-term artistic accumulation and experience, suggesting that while traditional methods had an advantage in creativity, they might fall short in efficiency and readability. The proposed AI layout design model, through DL technology, achieved the automatic extraction and combination of several design elements, not only improving design efficiency but also offering superior solutions in terms of visual aesthetics and functionality. Han et al. (2023) [57] proposed a neural style transfer method, applying an artist's style to any image by using CNNs to extract content and style features and then combining them to generate new images. Unlike this study, Han et al. 's research focused on style transformation rather than automated layout design. Although both studies leveraged the feature extraction capabilities of CNNs, this study further applied this capability to layout design in visual communication, addressing not only the aesthetic effect of images but also the effectiveness of information transmission and optimization of user experience. Further analysis revealed that the CNN model proposed in this study demonstrated strong adaptability in multi-layer convolutional feature extraction, similar to the CNN-based image recognition method proposed by Fu et al. (2024) [58], whose application in layout design proved the broad applicability of DL models in complex visual tasks. Moreover, through automatic optimization and parameter adjustment of the model, the AI layout design could meet diverse design needs and allow real-time adjustments based on user feedback, a dynamic optimization capability of great practical value in current visual communication design.

As an innovative application, the proposed AI layout design technology based on CNN demonstrates significant potential in layout design fields. By leveraging extensive learning from diverse layout design data to autonomously generate artistic layouts, AI layout design technology can alleviate designers' workloads and foster greater inspiration and creativity among designers. Keivanlou-Shahrestanaki et al. (2022) [59] employed ML techniques in their study and successfully generated artistic layout designs, demonstrating the potential of this technology in advertising design and brand communication. Tembhurne et al. (2022) [60] used DL technologies to analyze a large number of artworks, showcasing the machine's capability to generate artistic images and designs. These studies are closely related to AI layout design technology, providing credibility to the research outcomes. In this study's experiments, AI layout design methods achieve average ratings of 5.95 for overall comprehensive evaluation, 5.68 for text readability, and 5.74 for visual path rationality evaluation (using a Likert 7-point scale). These evaluation results markedly surpass traditional layout design methods, thereby validating the significant effectiveness of AI layout design technology in enhancing design efficiency and user experience. Overall, the results of this study not only validate the innovative capabilities of AI technology in the field of visual communication but also provide a new approach to design practice by combining DL and big data analysis to generate efficient and creative design solutions. Future research could further expand the application scope of this model, explore its potential in more art forms, and delve into the synergistic effects of different AI algorithms, to make a greater impact on the development of new media art.

## 5 Conclusion

### 5.1 Research contribution

New media art, as a burgeoning fusion of science and art, is increasingly capturing attention. AI-based layout design technology holds promising applications in the new media art field. The AI layout design technology rooted in CNN shows good effect and reliability in practical application. This technology has high accuracy and expressiveness in generating artistic images and designs, satisfying diverse user needs and expectations. This study promotes the deep integration of AI technology in the fields of art and design. By involving ML mechanisms in layout design processes, traditional design boundaries are broken, enabling automated generation and composition of design elements. This significantly improves design efficiency and provides designers with more creative inspiration and possibilities, promoting innovation and development in the art and design fields.

### 5.2 Future works and research limitations

This study explores the synergistic effects of AI technology in the evolution of new media art visual communication and validates its application potential in visual communication design practices through a CNN-based AI layout design method. However, despite achieving some positive outcomes, several challenges remain to be addressed in future research and applications. Firstly, the application of AI technology in visual communication design still faces the challenge of interdisciplinary knowledge integration. The development of AI technology requires the integration of knowledge from multiple fields, including computer science, art design, and cognitive psychology. Currently, the collaboration between different disciplines is not yet close enough, leading to certain limitations in the practical application of AI in design. Future research should focus more on interdisciplinary collaboration, facilitating the integration and sharing of knowledge to achieve a deeper fusion of technology and art, thereby further enhancing the practical application of AI technology in visual communication design. Secondly, although CNNs perform well in image feature extraction and layout design generation, they still face issues such as high computational costs and long training times when handling complex design tasks. Additionally, due to the diversity and complexity of design tasks, existing AI models often require extensive tuning and optimization to meet different design needs, which raises the technical demands of designers. Therefore, future research could explore more efficient algorithms and model optimization strategies to lower the barriers to using AI design tools, making them more accessible for practical design work. Finally, as AI technology continues to advance, ethical concerns are gradually coming to the forefront. AI-generated design works may spark controversies regarding intellectual property, originality, and cultural adaptability. Future research needs to explore the ethical standards of AI design in-depth while fostering technological innovation, ensuring that the application of technology aligns with societal moral standards and promotes the sustainable development of the new media art field. In summary, while the application of AI technology in new media art visual communication holds great promise, its further development requires overcoming challenges related to interdisciplinary collaboration, model optimization, and ethical standards. Future research should concentrate on these areas, driving continuous innovation and development of AI technology in visual communication design, and injecting more possibilities into the evolution of new media art.

## Funding

This research was supported by the Scientific Research Platform and Project of Guangdong Provincial Department of Education and Provincial Education Science Planning Project in 2023 (Higher Education Special Project), Project number 671. It was also supported by Research on the Development of Red Cultural and Creative Products in Museums under the New Model of Production-Education Integration with Seamless Connection of the “Four chains”in Vocational Education.

## Data availability statement

Data will be made available on request.

## CRediT authorship contribution statement

**Yan Zhao:** Writing – original draft, Visualization, Validation, Software, Resources, Project administration, Methodology, Investigation, Formal analysis, Data curation, Conceptualization.

## Declaration of competing interest

The authors declare that they have no known competing financial interests or personal relationships that could have appeared to influence the work reported in this paper.
